# Comparison of anaphylaxis epidemiology between urban and suburban pediatric emergency departments

**DOI:** 10.1186/s12887-023-03898-2

**Published:** 2023-02-18

**Authors:** Dhritiman Gurkha, Robert Podolsky, Usha Sethuraman, Kelly Levasseur

**Affiliations:** 1 Emergency Department, Dayton Children’s Hospital, Dayton, OH United States; 2 Department of Biostatistics, Beaumont Hospital, Royal Oak, MI United States; 3 Department of Emergency Medicine, Children’s Hospital of Michigan, Detroit, MI United States

**Keywords:** Pediatric anaphylaxis, Allergy, Urban, Suburban, Emergency department

## Abstract

**Background:**

Anaphylaxis is a serious allergic reaction that is rapid in onset and may cause death. To date, there are no published data on epidemiology of pediatric anaphylaxis in Michigan. Our objective was to describe and compare the time trends in incidence of anaphylaxis in urban and suburban populations of Metro Detroit.

**Methods:**

We performed a retrospective study of Pediatric Emergency Department (ED) anaphylaxis visits from January 1, 2010, to December 1, 2017. The study was conducted at 1 suburban ED (SED) and 1 urban ED (UED). We identified cases using an International Classification of Diseases (ICD) 9 and 10 query of the electronic medical record. Patients were included if they aged 0–17 years and met the 2006 National Institute of Allergy and Infectious Disease and the Food Allergy and Anaphylaxis Network diagnostic criteria for anaphylaxis. The anaphylaxis rate was calculated as the number of detected cases divided by the total number of pediatric emergency room visits for that month. Anaphylaxis rates were compared between the two EDs using Poisson regression.

**Results:**

A total of 8,627 patient encounters had ICD codes for anaphylaxis, of which 703 visits fulfilled the inclusion criteria and were used in subsequent analyses. Overall, the incidence of anaphylaxis was more common in males and in children < 4 years of age in both centers. Although the total number of anaphylaxis related visits was higher at UED over the eight-year time frame for this study, the anaphylaxis rate (cases per 100,000 ED visits) throughout the study was higher at the SED. While the observed anaphylaxis rate at UED was 10.47 – 162.05 cases per 100,000 ED visits, the observed anaphylaxis rate at SED was 0 – 556.24 cases per 100,000 ED visits.

**Conclusion:**

Pediatric anaphylaxis rates differ significantly between urban and suburban populations in metro Detroit EDs. The rate of anaphylaxis related visits to the ED has significantly increased over the past 8 years in the metro Detroit area, with significantly higher rise in suburban compared to urban ED. More studies are needed to explore the reasons for this observed difference in increase rates.

**Supplementary Information:**

The online version contains supplementary material available at 10.1186/s12887-023-03898-2.

## Background

Anaphylaxis is a serious allergic reaction that is rapid in onset and requires timely interventions [1]. The diagnosis of anaphylaxis is based on clinical criteria established in 2006 by the National Institute for Allergy and Infectious Disease (NIAID) and Food Allergy and Anaphylaxis Network (FAAN) (Appendix Item 1) [2, 3]. Anaphylaxis accounts for a significant number of emergency department (ED) visits and the rate has been increasing over the last 2 decades both in adults and children [4–6]. Pediatric emergency department (PED) visits anaphylaxis incidence in children increased from 3944 per million person-years in 2008 to 4510 per million person-years in 2016, (IRR 1.14; 95% CI,1.02–1.27) [4].

During 2005–2014, Motosue et al. also noted 196% increase in ED anaphylaxis incidence across the United States, especially among children aged 5–17 years (*P* < 0.001) [6]. The most common causes for anaphylaxis across all age groups include food (33%), stings (19%) and medications (14%) [7, 8]. In children, food allergies account for most cases of anaphylaxis [8]. The rates of food induced anaphylactic reaction leading to an ED visit range from 1 to 70 per 100,000 per year [1]. Children living in the inner city have a higher prevalence of allergies with greater asthma and anaphylaxis morbidity than the general population [9, 10]. Our objectives were to compare the urban/suburban differences in the epidemiology and associated risk factors for anaphylaxis related visits to two large EDs located in a Midwestern state.

## Methods

### Design and location

**Fig. 1:**
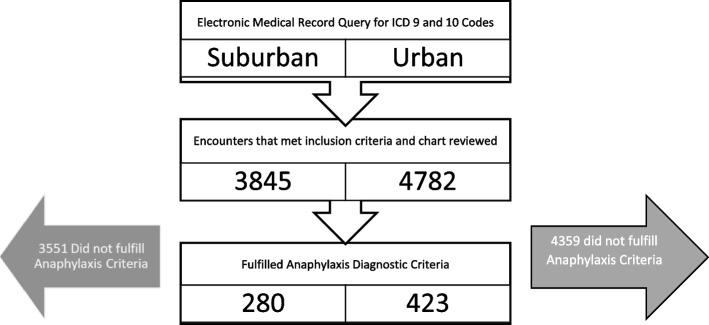
Anaphylaxis Study Flowsheet. Exclusion criteria: Patients > 17 years of age and those who did not meet the definition of anaphylaxis or had no encounter note in the chart were excluded.

We performed a retrospective chart review from January 1, 2010, to December 1, 2017, of pediatric anaphylaxis visits (Fig. 1). The study was conducted at 2 pediatric emergency departments within different hospital systems that were in urban and suburban areas of Metro Detroit. The urban ED (UED) is a free-standing level 1 Children’s hospital and trauma center with more than 85,000 annual visits. The Suburban PED (SED) is a level 2 pediatric trauma center located within a large adult ED with 23,000 annual ED visits.

### Population

#### Inclusion criteria

Patients aged < 18 years who were evaluated in the two EDs during the study dates and met the definition of anaphylaxis were enrolled in the study.

#### Exclusion criteria

Patients who did not meet the definition of anaphylaxis or had no encounter note in the chart were excluded.

#### Study definitions

Anaphylaxis was defined using the criteria outlined in the Second Symposium on the Definition and Management of Anaphylaxis established by NIAID/FAAN (Appendix Item 1) in 2006 [2].

#### Study data

Anaphylaxis cases were identified from the electronic medical record (EMR) with a query using admitting and/or discharge diagnoses with the International Classification of Diseases—Ninth Edition (ICD-9) codes 995.XX (allergic reactions) and 989.5 (sting or venom reaction) in addition ICD10 code T78.2 (anaphylactic reaction or shock).

Specific ICD 9 and 10 codes are listed in the appendix item A 2.

Identified electronic medical charts were screened by the PI at each site using the standardized anaphylaxis definition of NIAID/FAAN criteria to determine inclusion criteria. Cases were excluded from final analysis if they had only one system involved (eg considering skin and mucus membrane involvement as two systems, Data was then extracted from all medical charts of patients meeting inclusion criteria. Data was extracted using a combination of the ED, EMS and any prearrival notes as applicable. A common data collection sheet on Excel (version: 2021) was created (Appendix  3).

The following data were recorded into an Excel file: demographics, chief complaint, systems involved at presentation (respiratory, mucocutaneous, gastrointestinal, cardiac), family and medical history, insurance type, medications given prior to ED arrival and in the ED and dispositions (admit vs discharge). Every effort was made to identify details on any missing data. But if data were absent after reviewing all the above sources of documents, it was deemed to have not been present. For eg, if a child presented with wheezing which was documented but a rash was not mentioned, it was presumed to not have occurred.

All data were extracted by a trained research assistant (one at each institution). Training for both research assistants was done by the PI of the primary institution. Monthly meetings were held between the respective PI of each institution and the research assistant to go over data entry and resolve any questions. Any differences in opinion between the site PIs were resolved by the third ED investigator (KL). Approximately 10% of all the charts were then checked by the PI at the primary institution (DG) for accuracy. If more than two errors were noted, the specific research assistant was asked to recheck all the entries. Both research assistants were medical students who were blinded to the objectives of the study.

### Statistical analyses

Demographic data were described using descriptive statistics. Characteristics of anaphylaxis cases were compared between hospitals using a two-sample t-test for age, Pearson’s chi-square test for categorical data with at least five observations per cell, and Fisher’s exact test for categorical data with less than five observations per cell. We summarized the number of anaphylaxis cases by month and year. We calculated the anaphylaxis rate as the number of detected cases divided by the total number of pediatric emergency room visits for that month. These monthly summarized data were then used for all subsequent analyses. We used Poisson regression to compare the anaphylaxis rates between SED and UED [11, 12]. The number of anaphylaxis cases was used as the dependent variable, and the total number of emergency room visits was included as an offset. We included hospital as a factor, study month as a continuous variable, and the interaction between hospital and study month as independent variables in the regression [11]. We evaluated the potential of overdispersion based on the scaled Pearson χ2 divided by the degrees of freedom [12]. This statistic showed some evidence of overdispersion (Scaled Pearson χ2/*df* = 1.32). As such, we compared the Poisson regression with a generalized linear model that used the negative binomial distribution.

While the scaled Pearson χ2 was closer to the degrees of freedom (Scaled Pearson χ2/*df* = 1.06), model fit statistics (Akaike information criterion, AIC, and Bayesian information criterion, BIC) did not change drastically relative to the Poisson regression model (AIC decreased by 3.72; BIC decreased by 0.47). We used Proc Genmod in SAS (Version 9.4) for all initial analyses. Based on the fit statistics, we used Poisson regression with a linear relationship between anaphylaxis risk and study month for the results [11, 12].

## Results

A total of 8,627 patient encounters had ICD codes for allergies. After chart review, majority of encounters were excluded as they did not meet the anaphylaxis diagnostic criteria. A total of 703 visits fulfilled the inclusion criteria and were used in subsequent analyses (Fig. 1). Specifically at SED, there were 255 unique patients: 234 of them were single visits while 17 had two visits and 4 of them had three visits.

Patient demographics of the visits from both EDs are presented in Table 1. Overall, the incidence of anaphylaxis was more common in males and in children < 4 years of age in both centers. The average age at SED and UED was 9.0 ± 5.7 years and 8.2 ± 5.2 years, respectively. The suburban and urban populations differed in race (*p* < 0.0001), age distribution (*p* < 0.02) and insurance types (*p* < 0.0001; Table 1). While there was higher proportion of urban children with history of allergies (p < 0.0001) and asthma as a comorbidity (*p* < 0.0068), family history of anaphylaxis was higher among the suburban population (*p* < 0.0001).

**Table 1:** Patient Demographics, personal and family history of atopic diseases

**Parameters**		**Suburban** **[*n* = 255], (%)**	**Urban** **[*n* = 390], (%)**	***P*-value**
**Age (years)**	0–4	78 (30.59)	132 (33.85)	0.1390
	5–9	52 (20.39)	101 (25.90)	
	10–14	74 (29.02)	98 (25.13)	
	15–17	51 (20.00)	59 (15.13)	
**Gender**	Male	154 (60.39)	231 (59.23)	0.7688
	Female	101 (39.61)	159 (40.77)	
**Race**	Caucasian	141 (55.73)	52 (13.33)	< 0.0001
	Black	55 (21.74)	292 (74.87)	
	Asian	22 (8.70)	1 (0.26)	
**Ethnicity**	Hispanic	9 (3.56)	18 (4.62)	0.513
**Family History**	Family History of anaphylaxis	66 (25.88)	3 (0.77)	< 0.0001
	History of Allergies	104 (40.78)	242 (62.05)	< 0.0001
**Personal History**	Asthma	81 (31.76)	172 (44.10)	0.002
	Eczema	34 (13.33)	81 (20.77)	0.016
	Allergic Rhinitis	3 (1.18)	20 (5.13)	0.008

Mucocutaneous was the most common system involved at both PEDs, but respiratory system involvement was nearly doubled in urban population. (Table 2). With regards to disposition, most patients were discharged from both EDs. However, admission rates were significantly higher amongst the urban cohort.

**Table 2:** Systems involved, Insurance type and Disposition at the encounter presentation

Systems Involved	Suburban	Urban	*p*- value
Mucocutaneous	205 (73.21)	394 (93.14)	< 0.0001
Respiratory	133 (47.50)	339 (80.14)	< 0.0001
Gastrointestinal (GI)	56 (20.00)	143 (33.81)	0.0001
Insurance type			< 0.0001
Private	218 (78.42)	110 (26.00)	
Public	60 (21.58)	298 (70.45)	
Uninsured	0 (0.00)	15 (3.55)	
Disposition			< 0.0001
Admitted	28 (10.00)	63 (14.89)	
Observation	1 (0.36)	176 (41.61)	

**Fig. 2:**
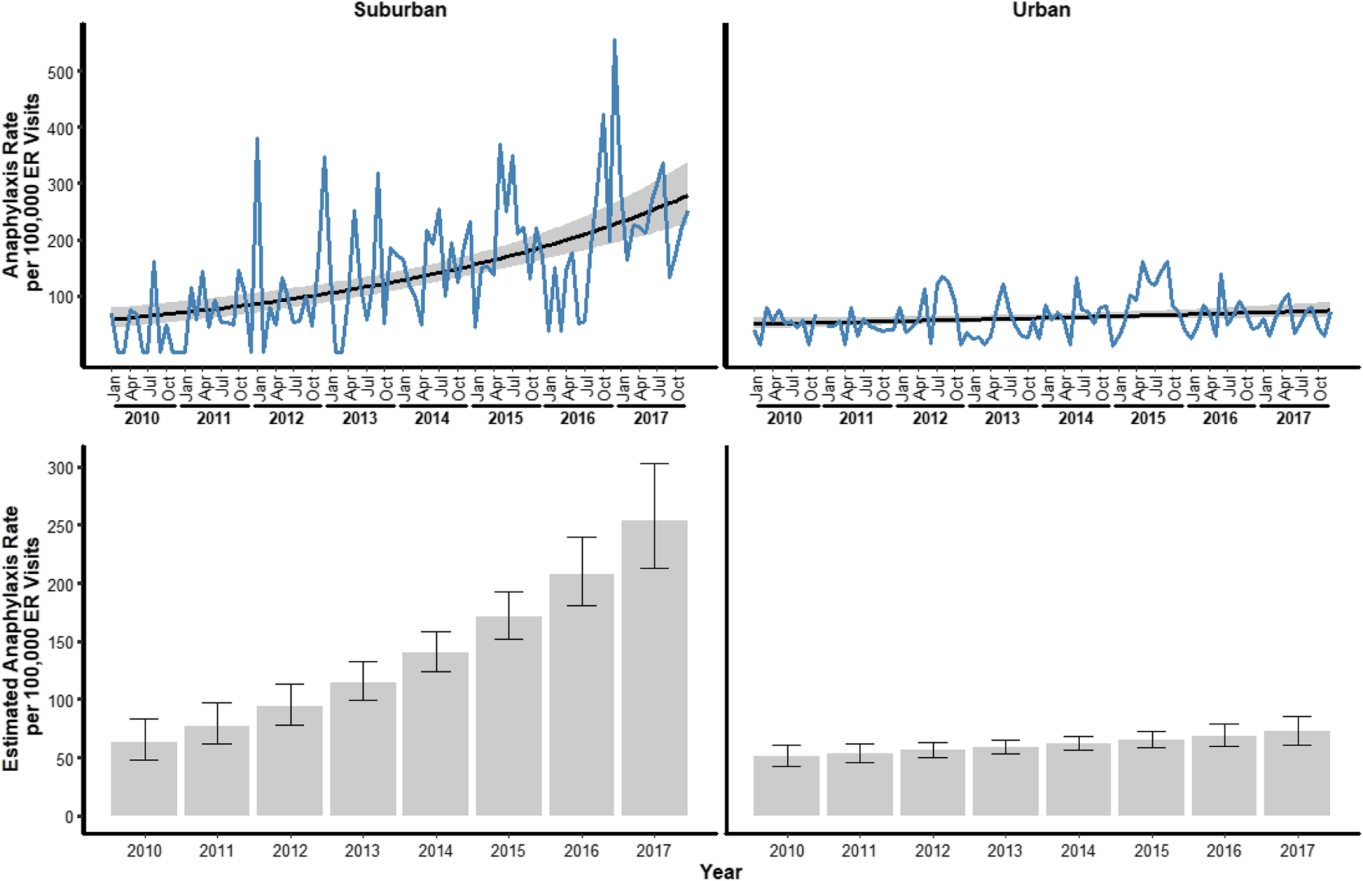
The relationship between anaphylaxis rate and study month for both centers (Suburban and Urban) over the eight-year study time frame. Top Panel- The observed monthly rate is plotted as the blue line. The line obtained from the statistical model and the confidence interval are shown in black with the grey band. Bottom Panel—The bottom plots show the model-based estimated anaphylaxis rate for each year.

Although the total number of anaphylaxis related visits was higher at UED over the eight-year time frame for this study, the anaphylaxis rate (cases per 100,000 ED visits) throughout the study was higher at the SED (Fig. 2). The change over time in the anaphylaxis rate differed significantly between SED and UED (*p* < 0.0001) (Fig. 2).

The observed anaphylaxis rate at suburban was 0 – 556.24 cases per 100,000 ED visits, while the observed anaphylaxis rate at urban was 10.47 – 162.05 cases per 100,000 ED visits. The change over time in the anaphylaxis rate differed significantly between suburban and urban (*p* < 0.0001). As such, we compared suburban and urban at the beginning of the study, with the initial estimated rate for suburban being 57.86 cases per 100,000 ED visits (95% CI: 42.84 – 78.15) and for urban being 49.79 cases per 100,000 ED visits (95% CI: 40.86 – 60.66). These estimated rates did not differ significantly (*p* = 0.4129). However, the difference in the estimated rates increased over time, with January 2011 being the first time-period with a statistically significant difference in the estimated anaphylaxis rates (*p* = 0.0500; suburban rate per 100,000 ED visits: 70.54; 95% CI, 54.89 – 90.66; urban rate: 52.32; 95% CI, 44.48 – 61.54). The final estimates were 277.88 cases per 100,000 ED visits (95% CI: 228.15 – 338.45) at suburban and 73.78 cases per 100,000 ED visits (95% CI: 61.38 – 88.68) at urban, with the difference in these rates being statistically significant (*p* < 0.0001).

There was no difference in the initial period’s estimated rates between the two area EDs.

However, the difference in the estimated rates increased over time, with January 2011 being the first time-period with a statistically significant difference in the estimated anaphylaxis rates per 100,000 ED visits. There was a significant difference in the final estimates of cases rates between the two EDs per 100,000 visits (Fig. 2). The percent increase in cases over the study period for SED was 379% while for UED it was 48%.

## Discussion

Our report summarizes the pediatric anaphylaxis visit rates at an urban and suburban PED location in a Midwestern state and compares the regional trends. We found an increase in anaphylaxis related visits to the PED during the study period, consistent with other studies [2, 3, 5]. However, unlike previous studies, we found significant differences in the incidence of pediatric anaphylaxis visit rates between the suburban and urban populations with significantly higher rates in suburban children.

Previous studies have reported increases in rates of anaphylaxis visits to the PED. In Illinois during 2008–2012, Dyer et al. found increased rates of anaphylaxis related ED visits and hospitalizations from 6.3 to 17.2 per 100,000 [10]. Similarly, Joshua et al. noted an increase in incidence of food related anaphylaxis in their retrospective study from 37 children’s hospital during 2007–2012 [13]. We found significantly higher rates of anaphylaxis related visits in children when compared to previous reports [13]. Given the consistent population-based trends of increasing rates of anaphylaxis cases, it is important to determine if all areas of the country are affected equally and to investigate possible causes for this rise in pediatric anaphylaxis. Although not within the scope of our study, it may be valuable to examine statewide data to evaluate any existing pockets of increased anaphylaxis incidence or “hotspots” based on zip codes.

Contrary to other studies, we found both a significantly higher rate of anaphylaxis related visits at baseline and a higher rate of increase over time in the suburban PED compared to the urban one [10]. In Illinois, Dyer et al. noted that Asian children, children with private insurance and children from urban Chicago neighborhoods had highest ED visit rates for anaphylaxis [10]. While the reason for our observed increase in suburban PED is unclear, Sakai-Bizmark et al. who compared urban with rural groups, suggest the Hygiene hypothesis as a possible explanation for rising rates of anaphylaxis [7]. This hypothesis theorizes that children who have fewer microbiologic challenges in childhood are more likely to develop anaphylaxis as exposure to certain infectious agents early in childhood lends protection against allergic reactions [7, 14]. Children exposed to fewer microbiologic challenges may lead to increased rates of atopy and potential allergic tendencies in the more affluent communities that may have better access to cleaning products and live in a more sterile environment.

Another possible explanation of these differences could be inequitable access to health care and financial resources between the suburban and urban groups. This corresponds to the demographic breakdown of general population when comparing the two cities where the respective EDs are located. The Royal Oak median household income was more than two and a half times that of the City of Detroit [15]. Approximately 36.4% of Detroit population lives in poverty, compared to 7% of Royal Oak [15].

### Limitations

This is a retrospective chart review and hence limited by the documentation available. Specifically, the percentage of patients with asthma in the suburban population may be under-reported if the past medical history was not elicited during the ED encounters.

The data reviewed did not have accurate charting of the atopy status of the patient or immediate family, which may potentially explain the possible differences in the incidence rates. A prospective study with preformed medical history questionnaire detailing the atopic history would provide appropriate information. We did not perform a kappa between the study team members and hence there is a possibility of interobserver variability in data extraction. However, through our periodic reviews of data, random chart checks and review of 10% of charts with corrections, we are confident in the accuracy of our data.

## Conclusion

In this study performed at two large tertiary children’s hospitals, there was a 379% increase in anaphylaxis rates over an eight-year period among suburban children as compared to 48% increase among the urban population. Future prospective studies that include geomapping would help confirm our findings and develop targeted interventions.

## Supplementary Information

**Additional file 1:** **Appendix Item 1.** NIAID/FAAN Anaphylaxis Diagnostic Criteria (Adapted from Sampson et al)^2^. **Appendix Item 2.** ICD 9 and 10 codes.

**Additional file 2:** **Appendix** **3.**

## Data Availability

Data no longer available owing to end of access period from IRB approval. Please try to contact Elizabeth.kring@beaumont.org for further information and assistance regarding data access.

## Abbreviations

ED: Emergency department
UED: Urban emergency department
SED: Suburban emergency department
