# Supplementary material for: Comparison of anaphylaxis epidemiology between urban and suburban pediatric emergency departments
Source: BMC Pediatr. 2023 Feb 18;23:85. doi: 10.1186/s12887-023-03898-2 (PMC9938607; doi:10.1186/s12887-023-03898-2)
Supplement: Supplementary file 1 — Additional file 1: Appendix Item 1. NIAID/FAAN Anaphylaxis Diagnostic Criteria (Adapted from Sampson et al)2. Appendix Item 2. ICD 9 and 10 codes. [file 12887_2023_3898_MOESM1_ESM.docx]

**Appendix**

| **Anaphylaxis is highly likely when any one of the following 3 criteria are fulfilled:** |
| --- |
| 1. Acute onset of an illness (minutes to several hours) with involvement of the skin, mucosal tissue, or both (generalized hives, pruritus or flushing, swollen lips-tongue-uvula) |
| AND at least one of the following: |
| a. Respiratory compromise (eg, dyspnea, wheeze-bronchospasm, stridor, reduced PEF, hypoxemia) |
| b. Reduced BP or associated symptoms of end-organ dysfunction (eg, hypotonia [collapse], syncope, incontinence) |
| 2. Two or more of the following that occur rapidly after exposure to a likely allergen for that patient (minutes to several hours): |
| a. Involvement of the skin-mucosal tissue (eg, generalized hives, itch-flush, swollen lips-tongue-uvula) |
| b. Respiratory compromise (eg, dyspnea, wheeze-bronchospasm, stridor, reduced PEF, hypoxemia) |
| c. Reduced BP or associated symptoms (eg, hypotonia [collapse], syncope, incontinence) |
| d. Persistent gastrointestinal symptoms (eg, crampy abdominal pain, vomiting) |
| 3. Reduced BP after exposure to known allergen for that patient (minutes to several hours): |
| a. Infants and children: low systolic BP (age specific) or greater than 30% decrease in systolic BP* |
| b. Adults: systolic BP of less than 90 mm Hg or greater than 30% decrease from that person’s baseline |

******Low systolic blood pressure for children is defined as less than 70 mm Hg from 1 month to 1 year, less than (70 mm Hg + [2 x age in years]) from 1 to 10 years, and less than 90 mm Hg from 11 to 17 years*

**Appendix Item 1: NIAID/FAAN Anaphylaxis Diagnostic Criteria (Adapted from Sampson *et al*)^2^**

| **ICD 9 Code** | **Diagnosis** |
| --- | --- |
| 995.3 | Allergy, unspecified not elsewhere classified |
| 995.27 | Other drug allergy(995.27) |
| 995.7 | Other adverse food reactions, not elsewhere classified |
| 995.61 | Anaphylactic reaction due to peanuts |
| 989.5 | Toxic effect of venom(989.5) |
| 995.29 | Unspecified adverse effect of other drug, medicinal and biological substance(995.29) |
| 995.64 | Anaphylactic reaction due to tree nuts and seeds |
| 995.66 | Anaphylactic reaction due to food additives |
| 995.2 | Unspecified adverse effect of unspecified drug, medicinal and biological substance |
| 995.1 | Angioneurotic edema not elsewhere classified |
| 995.6 | Anaphylactic reaction due to unspecified food |
| 995.65 | Anaphylactic reaction due to fish |
| 995.69 | Anaphylactic reaction due to other specified food |
| 995.67 | Anaphylactic reaction due to milk products(995.67) |
| 995 | Other anaphylactic reaction |
| 995.63 | Anaphylactic reaction due to fruits and vegetables |
| 995.62 | Anaphylactic reaction due to crustaceans |
| 995.4 | Shock due to anesthesia not elsewhere classified |
| 995.23 | Unspecified adverse effect of insulin(995.23) |
| 995.89 | Certain adverse effects, not elsewhere classified, other |
| 995.68 | Anaphylactic reaction due to eggs |
| **ICD 10 Code** | **Diagnosis** |
| T78.40XA | Allergy, unspecified, initial encounter |
| T78.40XD | Allergy, unspecified, subsequent encounter |
| T78.40XS | Allergy, unspecified, sequela |
| T78.49XA | Other allergy, initial encounter |
| T78.49XD | Other allergy, subsequent encounter |
| T78.49XS | Other allergy, sequela |
| T78.00XA | Anaphylactic reaction due to unspecified food, initial encounter |
| T78.00XD | Anaphylactic reaction due to unspecified food, subsequent encounter |
| T78.00XS | Anaphylactic reaction due to unspecified food, sequela |
| T78.01XA | Anaphylactic reaction due to peanuts, initial encounter |
| T78.01XD | Anaphylactic reaction due to peanuts, subsequent encounter |
| T78.02XA | Anaphylactic reaction due to shellfish (crustaceans), initial encounter |
| T78.02XD | Anaphylactic reaction due to shellfish (crustaceans), subsequent encounter |
| T78.03XA | Anaphylactic reaction due to other fish, initial encounter |
| T78.03XD | Anaphylactic reaction due to other fish, subsequent encounter |
| T78.04XA | Anaphylactic reaction due to fruits and vegetables, initial encounter |
| T78.04XD | Anaphylactic reaction due to fruits and vegetables, subsequent encounter |
| T78.04XS | Anaphylactic reaction due to fruits and vegetables, sequela |
| T78.05XA | Anaphylactic reaction due to tree nuts and seeds, initial encounter |
| T78.05XD | Anaphylactic reaction due to tree nuts and seeds, subsequent encounter |
| T78.05XS | Anaphylactic reaction due to tree nuts and seeds, sequela |
| T78.06XA | Anaphylactic reaction due to food additives, initial encounter |
| T78.06XD | Anaphylactic reaction due to food additives, subsequent encounter |
| T78.07XA | Anaphylactic reaction due to milk and dairy products, initial encounter |
| T78.07XD | Anaphylactic reaction due to milk and dairy products, subsequent encounter |
| T78.08XA | Anaphylactic reaction due to eggs, initial encounter |
| T78.08XD | Anaphylactic reaction due to eggs, subsequent encounter |
| T78.08XS | Anaphylactic reaction due to eggs, sequela |
| T78.09XA | Anaphylactic reaction due to other food products, initial encounter |
| T78.09XD | Anaphylactic reaction due to other food products, subsequent encounter |
| T78.09XS | Anaphylactic reaction due to other food products, sequela |
| T78.2XXA | Anaphylactic shock, unspecified, initial encounter |
| T78.2XXD | Anaphylactic shock, unspecified, subsequent encounter |
| T78.2XXS | Anaphylactic shock, unspecified, sequela |
| T80.51XA | Anaphylactic reaction due to administration of blood and blood products, initial encounter |
| T80.52XA | Anaphylactic reaction due to vaccination, initial encounter |
| T80.59XA | Anaphylactic reaction due to other serum, initial encounter |
| T80.59XD | Anaphylactic reaction due to other serum, subsequent encounter |
| T88.6XXA | Anaphylactic reaction due to adverse effect of correct drug or medicament properly administered, initial encounter |
| T88.6XXD | Anaphylactic reaction due to adverse effect of correct drug or medicament properly administered, subsequent encounter |

**Appendix Item 2: ICD 9 and 10 codes**
